# Up-regulated MCPIP1 in abdominal aortic aneurysm is associated with vascular smooth muscle cell apoptosis and MMPs production

**DOI:** 10.1042/BSR20191252

**Published:** 2019-11-12

**Authors:** Ming Xue, Gang Li, Dan Li, Zhu Wang, Lei Mi, Jingjing Da, Xing Jin

**Affiliations:** 1 Department of Vascular Surgery, Shandong Provincial Hospital affiliated to Shandong University, Jinan 250021, Shandong, China; 2 Department of Interventional Radiology, Weihai Municipal Hospital, Weihai 264200, Shandong, China; 3 Office of the Director, Weihai Municipal Hospital, Weihai 264200, Shandong, China; 4 Department of Clinical Laboratory, Weihai Municipal Hospital, Weihai 264200, Shandong, China; 5 Department of Interventional Medicine and Vascular Surgery, The Affiliated Hospital of Binzhou Medical University, Binzhou 256603, Shandong, China; 6 Department of General Surgery, Taian City Central Hospital, Taian 271000, Shandong, China; 7 Department of Biomedical Science, Guizhou University Medical College, Guiyang 550000, Guizhou, China

**Keywords:** Abdominal aortic aneurysm, apoptosis, MCPIP1, MMP, VSMCs

## Abstract

Abdominal aortic aneurysm (AAA) is often clinically silent before rupture characterized by extensive vascular inflammation and degenerative elasticity of aortic wall. Monocyte chemotactic protein-induced protein-1 (MCPIP1) exhibits anti-infllammatory and pro-apoptotic effects involved in atherogenesis. However, little is known about the expression and the contribution of MCPIP1 in AAA. In the present study, we collected clinical AAA specimens and constructed AAA mice model through Ang-II infusion, and found apparently increased MCPIP1 expression and severe inflammatory infiltration in AAA aortic membrane as evidenced by elevated levels of monocyte chemotactic protein 1 (MCP-1), interleukin 1 β (IL-1β) and NF-κB, as well as HE staining. The elasticity of aortic tunica media was impaired along with multiple apoptosis of vascular smooth muscle cells (VSMCs) in Ang-II-induced aneurysmal mouse. *In vitro* Ang-II administration of VSMCs induced MCPIP1 expression, accompanied by up-regulation of matrix metalloproteinase (MMP) 2 (MMP-2) and MMP-9, as well as enhancement of VSMCs proliferation and apoptosis, which may cause damage of intima–media elasticity. Silencing MCPIP1 reversed above effects to further restore the balance of proliferation and apoptosis in VSMCs. Overall, our data indicated that up-regulation of MCPIP1 may become a promising candidate for the diagnosis of AAA, and specific knockdown of MCPIP1 in VSMCs could inhibit VSMCs apoptosis and down-regulate MMPs to maintain vascular wall elasticity. Therefore, knockdown of MCPIP1 may serve as a potential target for gene therapy of AAA.

## Introduction

Human abdominal aortic aneurysm (AAA) is a frequent age-related aortic disease with six-fold higher incidence among males over 60 years and females over 65 years [1,2]. Most AAA are asymptomatic until rupture, which is the leading cause of the high mortality of AAA. Approximately 80% AAA patients will die on the way to hospital and 50% of those undergo a ruptured surgery [3]. Hence, novel effective biomarkers for AAA pre-diagnosis and screening are warranted to decrease the death rate. Currently, open surgical or endovascular repair is the only clinical strategy for AAA, but is limited by the aortic diameter, as the surgery shows fewer benefits for small aneurysms typically <5.5 cm [4]. Therefore, searching for novel therapeutic methods is imperative to reduce mortality of this disease. AAA is a multifactorial disease characterized by extensive vascular inflammation and degenerative elasticity of aortic wall caused by vascular smooth muscle cells (VSMCs) apoptosis reduced elastin production [5,6]. Thus, inhibiting apoptosis of VSMCs and alleviating inflammation are mainstream directions for biomarkers and therapeutic approaches of AAA.

Monocyte chemotactic protein-induced protein-1 (MCPIP1), also known as Regnase-1 and Zc3h12a, belongs to the evolutionary highly conserved MCPIP family containing four members numbered as 1–4 [7]. MCPIP1 is widely expressed in immune cells and is well known as a repressor of inflammation [8]. Recent reports pointed out that MCPIP1(−/−) knockout mice showed immune alterations such as augmented serum immunoglobulin levels and autoantibody production [9]. Theoretically, MCPIP1 is induced by both monocyte chemotactic protein 1 (MCP-1) and interleukin 1 β (IL-1β) in human monocyte-derived macrophages, then the signals are transduced through NF-kβ or MAPK pathway [10]. In turn, MCPIP1 could act as an RNAse for degrading MCP-1, IL-1β and its own mRNA to inhibit inflammatory responses in an ATP-mediated RNA helicase upframeshift 1 (UPF1)-dependent manner by recognizing stem-loop structures specifically at 30-untranslated regions of cytokine mRNAs [8,11]. Apart from that, MCPIP1 is recognized as a tumor suppressor due to its induction of apoptosis of tumor cells, for instance by selectively enhancing mRNA decay of antiapoptotic gene transcripts, including Bcl2L1, Bcl2A1, RelB, Birc3, and Bcl3. Mechanistically, MCPIP1 physically interacted with a stem-loop structure in the 3′ untranslated region of these transcripts through its PIN domain, causing mRNA destabilization [12,13]. However, limited attention has been directed toward the expression and the contribution of MCPIP1 in AAA. Yu et al. [14] reported that bone marrow deficiency of MCPIP1 led to severe systemic and multi-organ inflammation but paradoxically diminished atherogenesis in hematopoietic mice. As AAA exerts severe atherosclerotic damage of vascular wall, we speculate that MCPIP1 may be up-regulated in response to inflammatory infiltration to induce elasticity degeneration of aortic wall in AAA.

In the present study, we found the expression of MCP-1, MCPIP1 and IL1β were up-regulated in both AAA patients and Ang-II-induced aneurysmal mice suggesting an enhanced inflammation response. Further silencing of MCPIP1 reversed the up-regulation of matrix metalloproteinase (MMP) 2 (MMP2) and MMP9 along with the high apoptotic rate in VSMCs induced by Ang-II, indicating that up-regulated MCPIP1 in AAA is associated with VSMCs apoptosis and MMPs production.

## Materials and methods

### Patients

AAA specimens were collected from patients undergoing AAA repair operations (*n*=10) at the Department of Vascular Surgery, Shandong Provincial Hospital affiliated to Shandong University from January 2017 to December 2018. Patients with aortitis, connective tissue disorders, or ruptured aneurysm were excluded. During surgery, aortic tissues were collected from the largest portion of the aneurysm, which were routinely excised and discarded during repair. Blood samples as healthy control were collected from volunteers with matched ages (*n*=10) while tissue samples of healthy control were from donors with sudden accidental death (*n*=10), who have no aortic disease and registered body donation.

The present study was approved by the Medical Ethics Committee of Shandong Provincial Hospital affiliated to Shandong University and was conducted in accordance with the Declaration of Helsinki. Written informed consent was obtained from all participants.

Venous blood samples were collected into serum separating tubes, followed by centrifuged for 20 min at 2500×***g*** and serum was prepared into aliquots before frozen at −80°C. Control abdominal aortic tissue was collected from ten age-matched organ donors without aortic aneurysm, dissection, coarctation or previous aortic repair. Periaortic fat and intraluminal thrombus were excluded, and samples were rinsed with phosphate buffered saline (PBS). Samples were snap-frozen and stored at −80°C for protein analysis. The serum samples were further used for enzyme-linked immunosorbent assay (ELISA). The mRNA or protein was isolated from blood and tissue sample in each case for further measurement.

### ELISA

Serum samples were diluted and applied into pre-coated plates. The quantitative sandwich ELISA was carried out to estimate the amount of target factors in plasma using Rat IL-1β ELISA Kit (Cat# E-EL-R0012c), Rat MCP-1 ELISA Kit (Cat# E-EL-R0633c), Rat NF-κB p65 (Nuclear Factor κB p65) ELISA Kit (Cat# E-EL-R0674c), Human IL-1β ELISA Kit (Cat# E-EL-H0149c), Human MCP-1 ELISA Kit (Cat# E-EL-H0020c) and Human NF-κB p65 ELISA Kit (Cat# E-EL-H1388c) according to the manufacturer’s directions. All ELISA kits were purchased from Elabscience Biotechnology Co., Ltd (Wuhan, China).

### Ang-II infusion mice model

Twenty 8-week-old mice were purchased from Animal Center of Jinan Pengyue Laboratory Animal Breeding Co., Ltd (Shandong, China) and randomly divided into the following two groups: saline infusion alone as sham (*n*=10) and Ang II infusion (*n*=10). Osmotic minipumps (Model 2004, Durect Corporation, Cupertino, CA, U.S.A.) were implanted into the mice to continuously deliver Ang-II (Sigma–Aldrich, St. Louis, MO) subcutaneously at a dose of 1000 ng/kg/min or saline vehicle for 28 days, as previously described [14]. The diameter of abdominal aorta was measured by ultrasonography (Siemens, Munich, Germany) on days 0, 7, 14 and 28 at Department of Ultrasound, Shandong Institute of Medical Imaging, and the aneurysm rate was calculated. All animal experiments were carried out according to the Institutional Animal Care and Use Committee Guidelines of Shandong Provincial Hospital affiliated to Shandong University. The experiments were mainly carried out in Central Laboratory of Shandong Provincial Hospital affiliated to Shandong University.

### Hematoxylin–Eosin staining

Mice aneurysmal tissues were dehydrated using a graded ethanol series of 70% for 2 h, 80% overnight, 90% for 2 h and 100% for 2 h, followed by post-fixed in dimethylbenzene for 30 min and kept in dimethylbenzene-paraffin for 2 h at 60°C before embedding in a metal frame. Sections (4 μm) were cut and placed on to slides to dry in a 70°C chamber for 40 min. Sections were dewaxed using ethanol and soaked into PBS for further experiments.

The sections were soaked in sequence with Hematoxylin (Servicebio, Wuhan, China) for 5 min, water for 5 min, and 1% hydrochloric ethanol for 3 s at room temperature, respectively, followed by rinsing with water for 20 min. After staining with Eosin for 3 min, the sections were dehydrated using a graded ethanol series of 75% for 2 min, 85% for 2 min, 95% for 2 min, and twice 100% for 5 min, and subsequently permeabilized twice with xylene for 10 min. The stained tissues were then mounted with resinene and photographed under a microscope (DP73; Olympus, Tokyo, Japan).

### Immunohistochemical analysis

The sections were deparaffinized, rehydrated, and incubated with methanol containing 0.3% H_2_O_2_ to block endogenous peroxidase, followed by autoclaving at 121°C for 10 min in citrate buffer (10 mM sodium citrate, pH 6.0) for antigen retrieval. Thereafter, the sections were coated with normal goat serum and immunohistochemically stained using streptavidinbiotin–peroxidase complex (SABC) technique. Briefly, sample sections were incubated with rabbit anti-MCPIP1 polyclonal antibody (1:100 diluted) (ab97910, Abcam, Cambridge, MA, U.S.A.), IgG control (Rabbit IgG-Isotype control 08-6199, Thermo Fisher Scientific, Massachusetts, U.S.A.) overnight at 4°C followed by staining with UltraSensitive™ SP (Mouse/Rabbit) IHC Kit (Maixin Biotech, Fuzhou, China) strictly according to the instruction manual. Diaminobenzidine (DAB, DAKO, Copenhagen, Denmark) was used as the chromogen, and nuclei were stained with Hematoxylin.

### Elastica van Gieson and Sirius Red staining

After deparaffinage and rehydration, aneurysmal sections were stained with Sirius Red or Elastica van Gieson (EVG) (both from Servicebio, Wuhan, China) following the manufacturer’s instructions. Images of each section were captured using a light microscope (OLYMPUS). The thicknesses of elastin lamellae and aortic wall were measured at ten evenly distributed positions on each aortic ring, and an average value was given. The extent of vascular injury was evaluated by the ratio of thickness of elastin and/or aortic wall.

### Tunel staining

Aneurysmal sections were permeabilized in 0.1% Triton X-100 for 8 min. After washing with PBS for three times, sections were blocked with 3% H_2_O_2_ at room temperature for 10 min. TUNEL reaction was performed using the *In Situ* Cell Death Detection Kit (Roche, Basel, Switzerland) strictly according to the manufacturer’s instructions. Thereafter, sections were visualized by DAB (DAKO) and stained with Hematoxylin for 3 min. Cell apoptosis was photographed under a microscope (OLYMPUS).

### Rat cell isolation, culture and drug treatment

The SD rats were purchased from Animal Center of Jinan Pengyue Laboratory Animal Breeding Co., Ltd (Shandong, China). All animal experiments were carried out according to the Institutional Animal Care and Use Committee Guidelines of Shandong Provincial Hospital affiliated to Shandong University. Adventitia was removed from adult SD rats, and the loose connective tissue was shaved completely.

VSMCs were isolated from the thoracic aorta of male SD rats (100–150 g) based on previous reports [15,16]. In brief, the animal was put into an anesthesia chamber and anesthetized for ∼5 min. After it stopped motor activity and the blinking rate became infrequent, it was killed by cervical dislocation. The dissected femurs and tibias were set in 70% isopropanol for a few seconds and transferred to DMEM (Gibco, Grand Island, NY, U.S.A.). The marrow of bone was flushed into a 50-ml tube by inserting the needle into one open end of the bone. The cells from marrow were resuspended and the bone debris and blood aggregates were removed through a 70-μm cell strainer. After centrifugation at 200×***g***, 4°C for 5 min, cells were cultured in 25 ml MSC medium (DMEM containing 10% fetal bovine serum (FBS) and 1% Pen-Strep) in a 37°C and 5% CO_2_ incubator for 1–2 weeks. Medium was changed every 2–3 days. The purity of VSMCs was approximately 90% assessed by cell immunostaining with anti-smooth muscle α-actin antibody (1:100 diluted, ab5694, Abcam, Cambridge, MA, U.S.A.). Only VSMCs between the third and fifth passages were used for experiments. VSMCs were grown in Roswell Park Memorial Institute-1640 (RPMI-1640, Gibco, Grand Island, NY, U.S.A.) supplemented with 10% FBS (HyClone, Logan, UT, U.S.A.) and streptomycin/penicillin (100 U/ml) at 37°C in an atmosphere of 5% CO_2_. VSMCs were exposed to 1 μmol/l Ang-II for 24 h for subsequent experiments, and untreated cells served as control.

### Flow cytometric analysis of cell cycle

Cells in each group were trypsinized and fixed with 70% ethanol, followed by washing with PBS. Cells were stained in 1 ml DNA Staining solution containing 10 μl Permeabilization solution (Cat #. CCS012, Multisciences (LIANKE) biotech, Co., Ltd, Hangzhou, China) for 30 min at RT in the dark and then analyzed immediately with a flow cytometer (Becton Dickinson, Franklin Lakes, U.S.A.). The percentage of cells across cell cycle was obtained using CellQuest software.

### Flow cytometric analysis of cell apoptosis

According to the protocol of the Annexin V-FITC/PI apoptosis kit (Cat. # AP101, MultiSciences, Hangzhou, China), the labeled cells were resuspended in 500 μl apoptosis positive control solution and mixed sequentially with 5 μl Annexin V-FITC and 10 μl PI, followed by flow cytometry.

### siRNA interference

Two siRNAs for MCPIP1 were designed synthesized by GenePharma Co., Ltd (Shanghai, China). Sequences were as follows: MCPIP1-siRNA-1: 5′-GCUACGAU GACCGAUUCAUUU-3′; MCPIP1-siRNA-2: 5′-GAGAGCCAGAUGUCAGAAUU U-3′; negative control: 5′-UUCUCCGAACGUGUCACGUTT-3′.

VSMCs were seeded and maintained in six-well plates at a density of 5 × 10^5^ per well until it reached 50% confluence. Then the two MCPIP1 siRNAs and NC were transfected into cells, respectively, using the siPORT Lipid Kit (Ambion, Austin, Texas, U.S.A.) strictly according to the manufacturer’s instructions. Cells were harvested at 2 days after transfection for subsequent experiments.

### EdU staining

VSMCs with respective treatment were collected at 48 h of transfection. EdU staining was conducted using EdU DNA Proliferation *in vitro* Detection (RiboBio, Guangzhou, China) according to the manufacturer’s protocol.

### Immunofluorescence staining

VSMCs were grown on coverslips at a density of 2 × 10^4^ per well with appropriate treatment and fixed in 4% formaldehyde for 30 min. After washing with PBS for three times, cells were permeated with 1 ml Triton X-100 at room temperature for 20 min. Thereafter, cells were incubated with primary antibodies against MCPIP1 (1:200 diluted, ab97910, Abcam, Cambridge, MA, U.S.A.), α-SMA (1:200 diluted, ab32575), rabbit IgG-Isotype Control (Alexa Fluor® 647) (1:500 diluted, ab199093) and rabbit IgG Isotype control (FITC) (1:100 diluted, ab223339) at 4°C overnight and subsequently incubated with FITC/Alexa Fluor® 647-labeled goat anti-rabbit IgG (H+L) secondary antibody (1:1000 diluted, ab6717/ab150115, Abcam, Cambridge, MA, U.S.A.) at a dilution of 1:200 for 2 h at 37°C. Unbound antibodies in each step were removed with PBS for three times. After staining with 4′,6-diamidino-2-phenylindole (DAPI) for 10 min and a final rinse with PBS, the coverslips were mounted inversely on to slides with 95% glycerol and observed under a fluorescence microscope (Olympus Corporation, Japan) and photographed.

### Western blot

Total proteins were extracted by lysing tissues or cells with lysis buffer (Beyotime, Haimen, China) according to the manufacturer’s directions. The protein concentration was detected using a Pierce™ BCA Protein Assay Kit (Thermo Fisher Scientific, Massachusetts, U.S.A.). A total of 40 μg proteins from each sample was resolved by SDS/polyacrylamide gel electrophoresis (PAGE) and transferred on to polyvinylidene fluoride (PVDF) membranes (Millipore, Bedford, MA, U.S.A.) at 300 mA for different times. The membranes were then blocked with 5% non-fat milk overnight and probed with primary antibodies against MCPIP1 (1:3000 diluted, ab97910, Abcam; transferred for 60 min), MCP-1 (1:2000 diluted, ab9669, Abcam; transferred for 25 min), IL-1β (1:500 diluted, ab2105, Abcam; transferred for 25 min), NF-κB (1:1000 diluted, ab16502, Abcam; transferred for 55 min), MMP-2 (1:5000 diluted, ab216433, Abcam; transferred for 65 min), MMP-9 (1:1000 diluted, ab38898, Abcam; transferred for 90 min) and GAPDH (1:1000 diluted, ab9485, Abcam) at 4°C overnight, followed by incubation with secondary anti-rabbit IgG-HRP antibody (1:20000 dilution, BOSTER, Wuhan, China) or anti-mouse IgG-HRP antibody (1:20000 dilution, BOSTER) at 37°C for 40 min. The target bands were visualized by enhanced chemiluminescence (ECL) solution (Millipore, Massachusetts, U.S.A.) and were analyzed by Gel-Pro-Analyzer software (Bethesda, MD, U.S.A.).

### RT-PCR

Total RNA of each sample was extracted with an RNA Fast Extraction Kit (BioTeke, Beijing, China) and was reverse-transcribed into cDNA using Bestar qPCR RT Kit (DBI®Bioscience, Ludwigshafen, Germany). One microliter of cDNA was amplified with 10 μl Bestar® SybrGreen qPCR masterMix (DBI®Bioscience, Ludwigshafen, Germany) and 10 μl primers in an Stratagene Real-Time PCR System (Mx3000P, Agilent Technologies, California, U.S.A.) with the following cycling profile: initial denaturation at 95°C for 2 min, 30 cycles consisting of 94°C for 20 s, 58°C for 20 s, and 72°C for 20 s. The following primers were used: MCPIP1, 5′-TTCCTGCGTAAGAAGCCACTC-3′ (sense) and 5′-GAATCGGCAC TTGATCCCATAG-3′ (antisense); GAPDH, 5′-TGTTCGTCATGGGTGTGAAC-3′ (sense) and 5′-ATGGCATGGACTGTGGTCAT-3′ (antisense) for human, MCPIP1, 5′-ACGAAGCCTGTCCAAGAATCC-3′ (sense) and 5′-AGTAGGGGC CTCTTTAGCCAC-3′ (antisense); β-actin, 5′-CATTGCTGACAGGATGCAGA-3′ (sense) and 5′-CTGCTGGAAGGTGGACAGTGA-3′ (antisense) for mouse, and MCPIP1, 5′-AGAAGCTGTGATGGGTCGTT-3′ (sense) and 5′-TGAGGTGCTGGGACTTGTAA-3′ (antisense); GAPDH, 5′-CCTCGTCTCATAGACAAGATGGT-3′ (sense) and 5′-CCTCGTCTCATAGACAAGATGGT-3′ (antisense) for rats. Relative mRNA expressions were calculated by 2^−ΔΔ*C*_T_^ method.

### Statistical analysis

Statistical analysis was performed with GraphPad Prism 5.0 software. All data are reported as mean ± standard deviation (SD). Differences between AAA group and control group were calculated with unpaired Student’s *t* test, and other differences between groups were compared with one-way analysis of variance (ANOVA). *P*<0.05 was considered to be statistically significant.

## Results

### MCPIP1-related inflammatory pathway is activated in AAA patients

**Figure 1:**
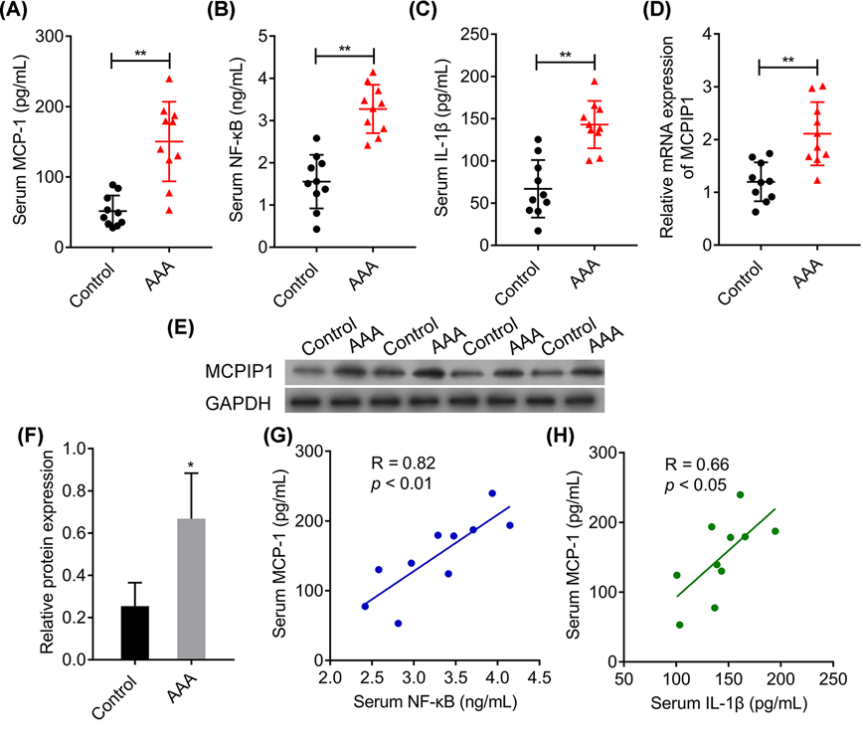
MCPIP1-related inflammatory pathway is activated in AAA patients (**A**) ELISA analysis of MCP-1 serum level, (**B**) ELISA analysis of NF-κB serum level, (**C**) ELISA analysis of IL-1β serum level. (**D**) RT-PCR analysis of MCPIP1 mRNA expression level. (**E**) Representative blot analysis of MCPIP1 expression are presented. (**F**) Corresponding densitometric analysis of MCPIP1 is shown as mean ± SD from three independent experiments. (**G**) Linear regression analysis between MCP-1 and NF-κB. (**H**) Linear regression analysis between MCP-1 and IL-1β. The above results are expressed as mean ± SD, and the error bars represent the SD of three independent experiments. *P<0.05, **P<0.01 *vs* control.

To identify MCPIP1-related inflammatory infiltration, we collected serum and tissue samples from AAA patients and healthy control for MCPIP1, MCP-1, IL1β and NF-κB detection. ELISA analysis showed that serum levels of MCP-1, IL1β and NF-κB were significantly elevated in comparison with healthy control (Figure 1A–C, *P*<0.01), and linear regression analysis showed that the expression of MCP-1 was positively correlated with NF-κB and IL1β in AAA patients (Figure 1G,H, *P*<0.01 and *P*<0.05 respectively). Besides, the mRNA level and protein level of MCPIP1 were both increased notably in AAA tissues compared with healthy control (Figure 1D–F, *P*<0.05). The above results revealed prominent inflammatory infiltration in aneurysm and suggested an interaction between MCPIP1 and the inflammation in AAA patients.

### MCPIP1-related inflammatory pathway is activated in Ang-II-induced aneurysmal mouse

**Figure 2:**
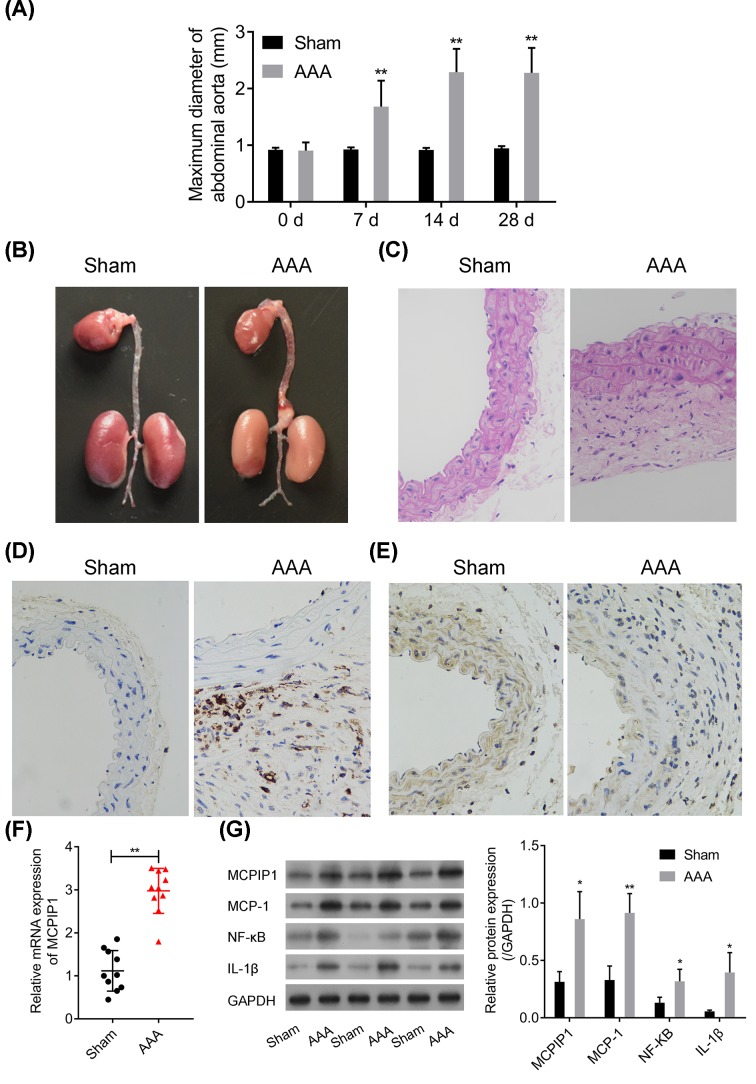
MCPIP1-related inflammatory pathway is activated in Ang-II-induced aneurysmal mouse (**A**) Ultrasonic detection of maximum diameter of abdominal aorta in mice with or without Ang-II administration. (**B**) Representative macroscopic appearance of the aorta in mice with or without Ang-II perfusion. (**C**) Representative photomicrographs of HE staining selected from paraffin-embedded sections of mice AAA and sham tissues. Eosinophils appeared to be red. Magnification 400× for all panels. (**D,E**) Representative photomicrographs selected from paraffin-embedded sections of AAA and healthy abdominal aorta tissues stained with antibody to MCPIP1 (D) and CD68 (E). Nuclei stained by Hematoxylin were observed to be blue or purple. The yellow, claybank particles in cytoplasm were target proteins. Magnification 400× for all panels. (**F**) RT-PCR analysis of MCPIP1 mRNA expression in AAA mice tissues. (**G**) Representative blots on MCPIP1, MCP-1, NF-κB and IL-1β in AAA mice tissues are presented and corresponding densitometric analysis is shown. All data are given as mean ± SD from three independent experiments. *P<0.05, **P<0.01 *vs* sham.

To confirm the MCPIP1-related inflammatory infiltration universally occurred in AAA, we generated an AAA mice model using Ang-II. A total of 11 mice models survived from 15 mice accounting for 72.4% survival rate. Macroscopic aneurysms (>50% dilatation of the abdominal aorta) developed in eight of eleven mice accounting for 72.7% (Figure 2A,B). The result of ultrasound assessment showed that the diameters of abdominal aorta in AAA mice after modeling for 14 and 28 days were apparently increased compared with that in sham group (Supplementary Figure S1). Abundant inflammatory cell infiltration was observed in AAA mice from HE staining and immunohistochemical CD68 staining on smooth muscle actins (Figure 2C,E). Furthermore, the expression of MCPIP1 was up-regulated significantly in aneurysm tissues compared with sham as evidenced by Western blot, immunohistochemical staining and RT-PCR assays (Figure 2D,F,G, *P*<0.01). Aneurysmal MCP-1, IL1β and NF-κB levels were coordinately elevated along with the inflammatory infiltration (Figure 2G, *P*<0.05), indicating that MCPIP1 is up-regulated in VSMCs in response to inflammatory infiltration in AAA progression.

### Impaired elasticity of aortic wall and enhanced apoptosis of VSMCs in Ang-II-induced aneurysmal mouse

**Figure 3:**
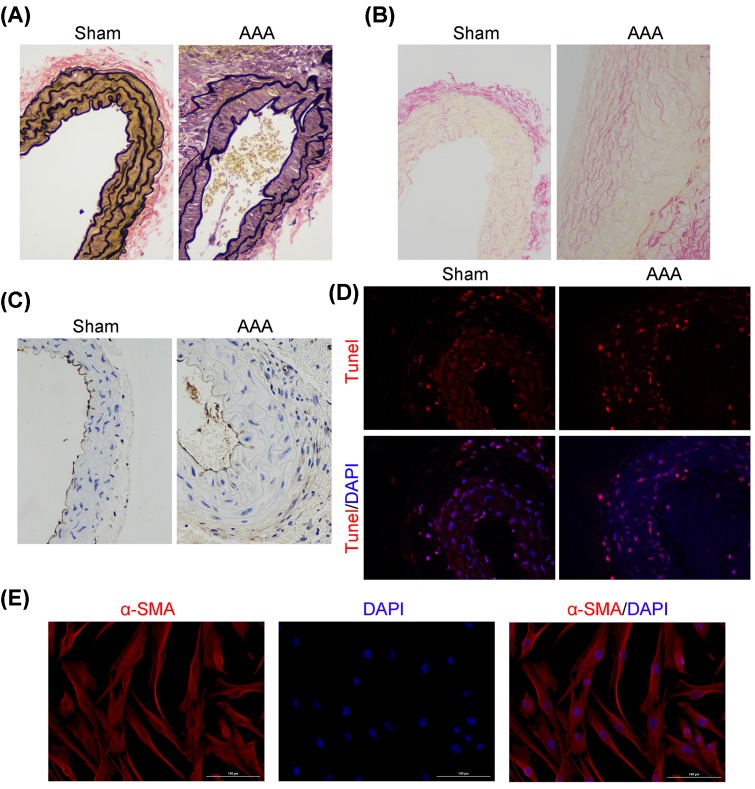
Impaired elasticity of aortic wall and enhanced apoptosis of VSMCs in Ang-II-induced aneurysmal mouse Representative photomicrographs of EVG staining (**A**), Sirius Red staining (**B**), immunohistochemical staining of CD31 (**C**) and Tunel staining (**D**) of two groups of mice (*n*=10 in each group used in pathological experiment). Magnification 400× for all panels. (**E**) The VSMCs were identified by immunofluorescence staining with anti-smooth muscle α-actin antibody. Nuclei labeled with DAPI were observed to be blue.

In order to investigate elastic changes of aortic wall in AAA mice, we performed EVG staining, Sirius Red staining, Immunohistochemistry and Tunel staining to evaluate the morphology of aortic elastic fibers and collagen fibers, as well as the survival of smooth muscle cells in AAA mice. As shown in Figure 3, the middle layer of aortic annulus in AAA mouse aortic aneurysm was incomplete with partially ruptured elastic annulus and lost wave shapes (Figure 3A). The red collagen fibers in adventitia aneurysm of AAA mice stained by Sirius Red dye were significantly declined, indicating a reduction in adventitial collagen deposition in aneurysms (Figure 3B). Although neovascularization was observed as evidenced by increased CD31 staining which is a vascular endothelial cell marker, the apoptotic VSMCs were increased obviously in AAA mice compared with sham (Figure 3C,D). The above results suggested an impaired elasticity of aortic wall in *Ang-II*-induced aneurysmal mouse. The VSMCs were further identified by immunofluorescence staining with anti-smooth muscle α-actin antibody and result showed the cells were successfully isolated, after validation by IgG control (Figure 3E and Supplementary Figure S2).

### Ang-II-induced MCPIP1 up-regulation destroys elasticity of aortic wall by increasing MMPs expression

**Figure 4:**
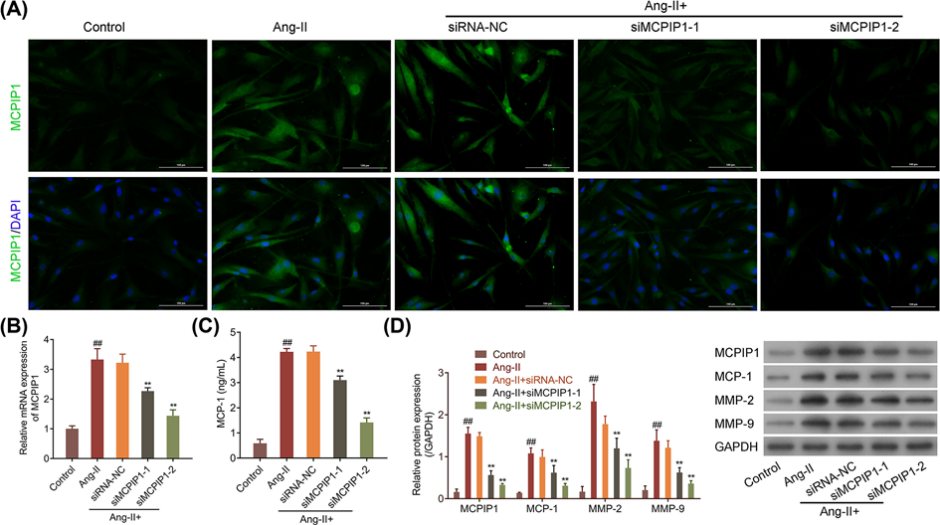
Ang-II-induced MCPIP1 up-regulation destroys elasticity of aortic wall by increasing MMPs expression VSMCs were exposed to Ang-II with or without siMCPIP1 transfection, followed by (**A**) Immunofluorescence staining with antibody to MCPIP1. Nuclei labeled with DAPI were observed to be blue. The green fluorescence in cytoplasm was target proteins. (**B**) RT-PCR detection on MCPIP1 mRNA expression level. (**C**) ELISA detection on MCP-1 level. (**D**) Western blot detection on MCPIP1, MCP-1, MMP-2 and MMP-9 expressions. Representative examples of images are shown. Experiments were done in triplicates for statistical significance, and the results are expressed as mean ± SD. ^##^*P*<0.01 *vs* control, **P<0.01 *vs* siRNA-NC.

To further identify the role of MCPIP1 in AAA, we simulated AAA for VSMC cells by Ang-II administration with or without MCPIP1 silencing to detect the expression of MCP-1 and MMPs. The expression of MCPIP1 in the cytoplasm of VSMCs with Ang-II treatment was tested and found significantly increased (Figure 4A,B, *P*<0.01), after it was validated by IgG isotype controls that only the cell nucleus was stained with DAPI (Supplementary Figure S2B). As shown previously in pathological samples and mice models, suggesting that Ang-II administration could mimic the status of VSMCs in AAA. Down-regulation of MCIPIP1 by specific siRNA reduced MCP-1 expression that raised by Ang-II in VSMCs (Figure 4C, *P*<0.01). Of note, the expression levels of MMP-2 and MMP-9 in Ang-II- treated VSMCs were 16.4-fold and 6.8-fold (Figure 4D, *P*<0.01) higher than control, which were decreased by 2.4-fold and 3.37-fold as compared with NC group respectively, suggesting that Ang-II-induced MCPIP1 up-regulation destroyed the elasticity of aortic wall by increasing MMPs expression to degrade elastic and collagen fibers.

### Ang-II-induced MCPIP1 up-regulation destroys elasticity of aortic wall induced by the imbalance between VSMC proliferation and apoptosis

**Figure 5:**
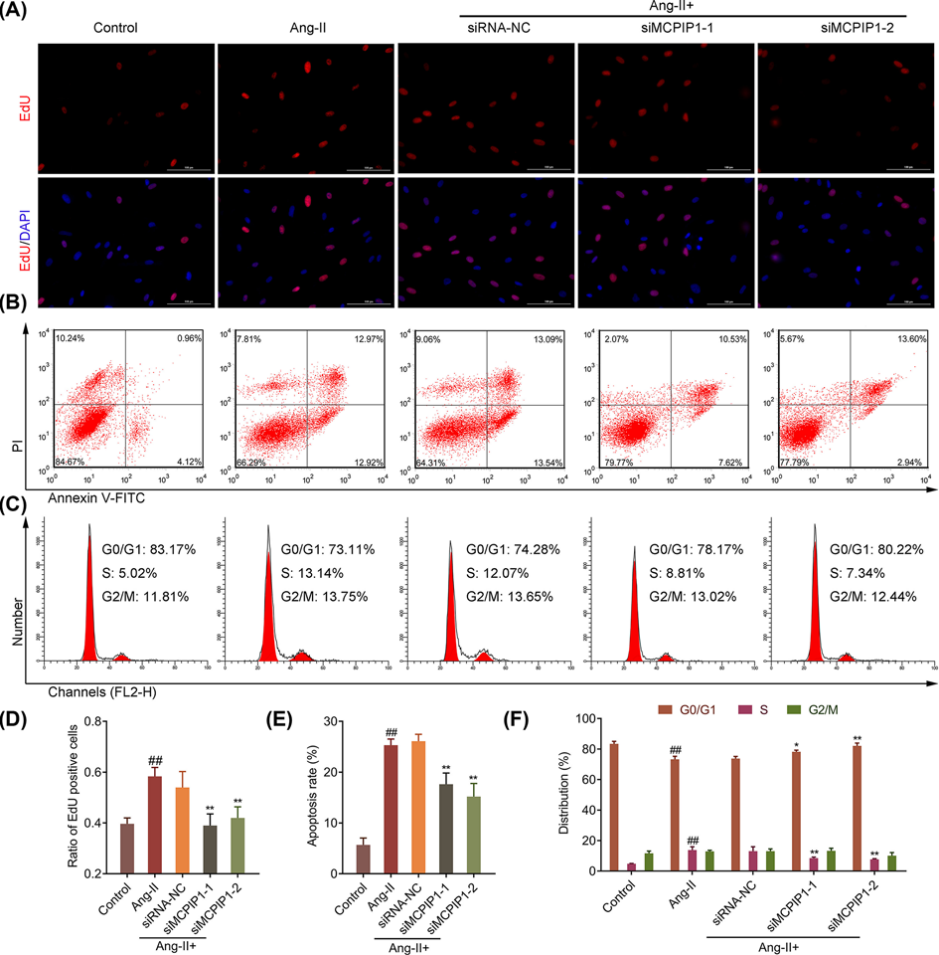
Ang-II-induced MCPIP1 up-regulation destroys elasticity of aortic wall induced by actuating VSMC apoptosis VSMCs were exposed to Ang-II with or without siMCPIP1 transfection, followed by (**A**) representative Edu staining photographs are shown. The red fluorescence indicates cells being in the proliferative phase. (**B**) Cell apoptosis was examined by Annexin V-FITC and Propidium Iodide staining followed by flow cytometry. The cells are characterized as early apoptotic (bottom right quadrant), late apoptotic (top right quadrant), necrotic (top left quadrant), and healthy cells (bottom left quadrant) based on the flow cytometry results. (**C**) Cell cycle distribution by flow cytometry analysis. Cells in each group were fixed and stained with Propidium Iodide, followed by flow cytometry analysis. (**D**) The total Edu positive cells are summarized in a bar chart. (**E**) The total apoptosis rate is summarized in a bar chart. (**F**) The percentages of cells at each cell cycle stage are summarized in a bar chart. Data in each group are expressed as mean ± SD from three independent experiments. ^##^*P*<0.01 *vs* control, *P<0.05, **P<0.01 *vs* siRNA-NC.

EdU and flow cytometry were employed to determine the effect of MCPIP1 on the proliferation and apoptosis of Ang-II-treated VSMCs. The results showed that the proliferation of VSMCs was significantly increased after Ang-II treatment, and the notable cytostatic effect appeared after silencing MCPIP1 in VSMCs (Figure 5A,C,D,F, *P*<0.01). In addition, the apoptotic rate in VSMCs with Ang-II treatment was 4.44-fold higher than that in control, which was repressed by 1.48-fold and 1.72-fold, respectively, after silencing MCPIP1 in comparison with NC (Figure 5B,E, *P*<0.01). Our data indicated that Ang-II-induced MCPIP1 up-regulation disturbs the imbalance between VSMCs proliferation and apoptosis, thereby destroying the elasticity of aortic wall.

## Discussion

MCPIP1 plays a key contributory role for AAA. Our data found the enhanced inflammatory symptoms in clinical AAA samples as described by the up-regulation of MCP-1, MCPIP1 and IL1β [17,18]. Interestingly, the control values of IL-1β in normal individuals in our study was approximately 70 pg/ml which displayed slightly higher levels compared with other study that the IL-1β average levels were 25 pg/ml, although they were roughly in a similar range (magnitude). We propose the slight difference of the data for the system error in different kits, as well as the characteristics and amount of patients, because the previous result regarding IL-1β levels were all obtained from investigated female [18]. It has been pointed out that bone marrow deficiency of MCPIP1 caused severe multi-organ inflammation but abrogated atherogenesis in hyperlipidemic mice, indicating MCPIP1 exhibits anti-inflammatory and pro-apoptotic effects involved in atherogenesis [14]. In our study, by contrast, the lack of MCPIP1 reduced the inflammation in an AAA mice model caused by Ang-II, which was inconsistent with previous evidence regarding MCPIP1 function. It has been reported that, thrombomodulin (TM) signals were predominantly localized to macrophages and VSMCs in human aneurysm specimens. In the mouse CaCl_2_-induced AAA model, deficiency of myeloid TM, but not VSMC TM, inhibited macrophage accumulation, attenuated proinflammatory cytokine and MMP-9 production, and finally mitigated elastin destruction and aortic dilatation [19]. Similarly, our result revealed that *in vitro* silencing MCPIP1 suppressed Ang-II-induced VSMC apoptosis and down-regulated Ang-II-upregulated MMP2 and MMP9 expressions, suggesting that the elevated MCPIP1 in AAA is associated with VSMCs apoptosis and MMPs production, and inhibition of MCPIP1 expression may contribute to the survival of VSMCs from inflammatory environment and maintain elasticity of aortic wall. MCPIP1 not only regulates the activation of innate and acquired immune cells via its degradation property, but also acts as an equilibrium gatekeeper to prevent immune system overactivation against inflammatory stimuli [9]. MCP-1, the most potent member in the CC chemokine family universally distributed in endothelial cells [20], macrophages [21] and fibroblasts [22], is capable of recruiting monocyte to inflammatory response sites and integrating polarized macrophages [23,24]. MCP-1 exhibits low or absent production in aortic smooth-muscle cells that enables VSMCs serve as a protective role against inflammation and proteolysis, but Yang et al. [25] showed that it was one of the most highly expressed chemokines in human AAA walls. The present study observed considerable infiltration of inflammatory cells in AAA patients and mice with elevated levels of MCP-1, IL-1β and NF-κB. Then the expression of MCPIP1 is up-regulated for degrading MCP-1 and IL-1β to save immune system from overactivation. Therefore, the elevated MCPIP1 level may become one of the promising indicators for AAA pre-diagnosis and screening.

Besides immune cells, degraded elasticity of aortic wall induced by VSMCs apoptosis plays an important role in AAA formation [26]. Recent studies have been identified that the significantly increased expression of MCPIP1 led to up-regulation of various apoptotic genes, which may trigger ischemic heart disease [27]. *In vitro* overexpression of MCPIP1 in cardiomyoblast H9C2 cells boosted cell death [7,28]. However, the effect of MCPIP1 overexpression on the apoptosis of VSMCs have rarely been reported. In our experiments, we found that silencing MCPIP1 reversed the pro-apoptotic effects of Ang-II in VSMCs. As we speculated that silencing MCPIP1 inhibits the VSMCs apoptosis, therefore ensure sufficient amount of VSMCs to produce elastin, and maintain the elasticity of aortic wall in inflammatory environment by inhibiting VSMCs apoptosis.

Except for apoptosis, VSMCs proliferation and apoptosis coexist at the early stage of aortic injury. VSMC cells exposed to various growth stimuli environment to promote the proliferation and further compensate for the cell loss induced by VSMCs apoptosis, which results in the imbalance of proliferation and apoptosis in the aorta [29]. In this study, silencing MCPIP1 not only inhibits the apoptosis of VSMCs, but also suppresses its overproliferation, thus maintaining the stability of VSMCs number in the aorta. Our *in vitro* data preliminarily identified the role of MCPIP1 in AAA through specific siRNAs targeting MCPIP1. We will further construct MCPIP1^−/−^ mice model to demonstrate the role of MCPIP1 in AAA formation by Ang-II infusion into MCPIP1^−/−^ mice.

MMPs include an arrangement of extracellular enzymes with proteolytic activities responsible for severing elastin, collagen, and fibrinogen to degrade the cellular matrix that is involved in multiple physiological processes, such as cancer metastasis, tissue remodeling and resorption [3]. MMP-2 and MMP-9 are therein the most intensively investigated MMPs reported in AAA [30,31]. Clinical researches point to the fact that the expression of MMP-2 and MMP-9 are increased with aneurysmal size in AAA patients [32]. MMP-9 reliably exhibits undetectable or extremely low level AAA repair. Patients with high levels of MMP-9 after surgery presents highly correlation with poor prognosis [33]. Our *in vitro* data showed that the levels of MMP2 and MMP9 were elevated apparently in VSMC with Ang-II treatment, which was consistent with previous studies. Additionally, the effect diminished by depletion of MCPIP1, indicating that MCPIP1 regulates MMP2 and MMP9 expression, and silencing MCPIP1 could maintain the elasticity of aortic vessel wall in inflammatory environment through down-regulating MMP2 and MMP9 expression.

In summary, our present study first revealed the up-regulation of MCPIP1 in AAA patients, which may become a promising target for AAA diagnosis. In addition, specific knockdown MCPIP1 in VSMCs contributes to maintaining vascular wall elasticity by reducing the apoptosis and down-regulation of MMPs in VSMCs. Therefore, knockdown MCPIP1 may serve as a potential target for gene therapy of AAA.

## Supplementary Material

Supplementary Figures S1-S2

## Abbreviations

AAA: abdominal aortic aneurysm
Ang-II: Angiotensin-II
DAB: diaminobenzidine
DAPI: 4′,6-diamidino-2-phenylindole
ELISA: enzyme-linked immunosorbent assay
EVG: Elastica van Gieson
FBS: fetal bovine serum
IL-1β: interleukin 1 β
MCPIP1: monocyte chemotactic protein-induced protein-1
MCP-1: monocyte chemotactic protein 1
MMP: matrix metalloproteinase
NF-κB p65: nuclear factor κB p65
PBS: phosphate buffered saline
TM: thrombomodulin
VSMC: vascular smooth muscle cell
