# Supplementary material for: Up-regulated MCPIP1 in abdominal aortic aneurysm is associated with vascular smooth muscle cell apoptosis and MMPs production
Source: Biosci Rep. 2019 Nov 12;39(11):BSR20191252. doi: 10.1042/BSR20191252 (PMC6851509; doi:10.1042/BSR20191252)
Supplement: Supplementary Figures S1-S2 [file BSR-2019-1252_supp.pdf]

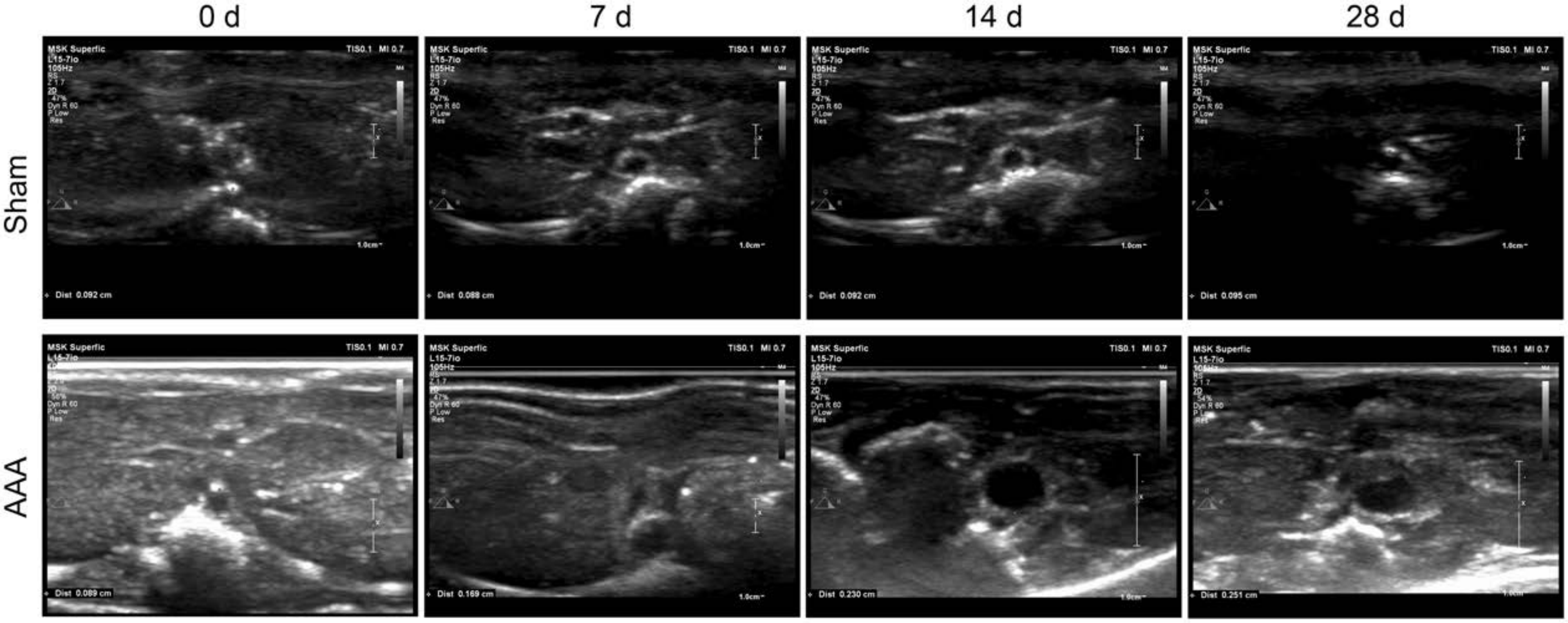

Fig. S1. Ultrasound assessment on aorta diameter changes. The difference of diameters of abdominal aorta between AAA and sham groups were determined and compared.

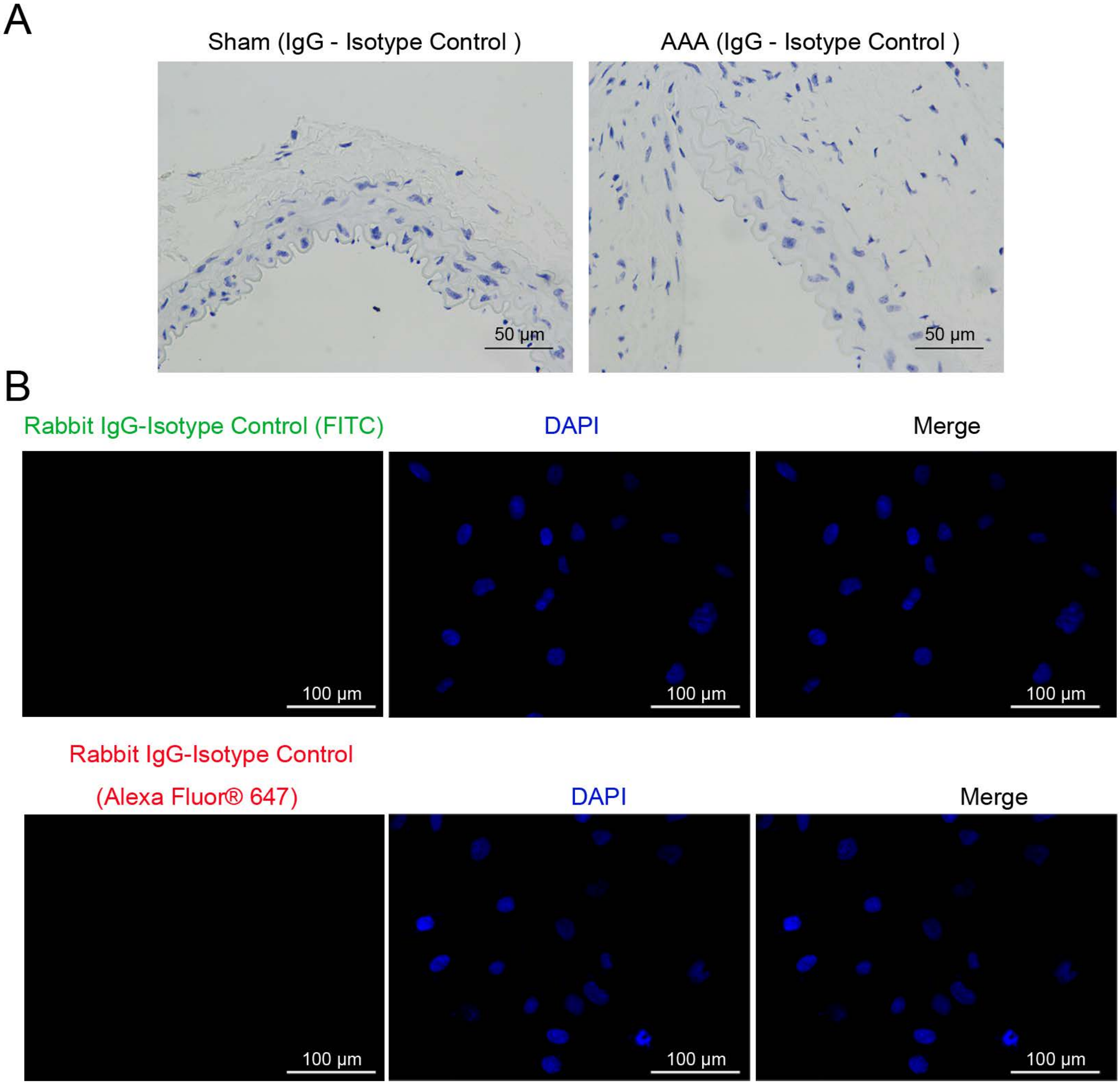

Fig. S2. Specificity of antibodies was validated by IgG-isotype control. (A) IgG-isoptype control for immunohistochemistry experiment. (B) IgG-isoptype control for Immunofluorescence staining. IgG-Isotype control of α-SMA (Alexa Fluor® 647) was labeled red while that of MCPIP1 (FITC) was green.
